# Formation evaluation of Abu Madi reservoir in Baltim gas field, Nile Delta, using well logs, core analysis and pressure data

**DOI:** 10.1038/s41598-023-46039-6

**Published:** 2023-11-06

**Authors:** Ahmed M. Metwally, Walid M. Mabrouk, Ahmed I. Mahmoud, Amr M. Eid, Mohammed Amer, Ahmed M. Noureldin

**Affiliations:** Geophysics Department, Faculty of Science, Cairo University, Giza, Egypt

**Keywords:** Geology, Geophysics

## Abstract

Baltim Eastern and Northern gas fields in the offshore Nile Delta have very high gas condensate accumulations. Therefore, the present research evaluates Abu Madi and Qawasim Formations and defines the petrophysical parameters for them using various data from five wells composed of wireline logs (gamma-ray, density, neutron, sonic, resistivity), core data, pressure data, and cross-plots. In the current study, the formations of the main reservoirs were evaluated qualitatively and quantitatively based on the petrophysical analysis to assess the production potential. Based on the lithological identification, the two main reservoirs (Abu Madi and Qawasim Formations) are composed of sandstone, calcareous shale, and siltstone. The main petrophysical parameters (Shale volume, effective porosity, net thickness, and fluid saturations) were mapped to track the areal petrophysical variations in the field. The results of the petrophysical analysis reveal that the main reservoirs are promising for the hydrocarbon potential with effective porosity of 18%, low shale content with an average value of about 21%, higher gas saturation of average value of nearly 58%, net reservoir thickness ranges from 25.5 to 131.5 m, net pay thickness (effective thickness) ranges from 6 to 61 m. Also, the conventional core analysis affirms that the main reservoirs are of good effective porosity with high horizontal and vertical permeability values. There is a good match between the well-log results and the pressure data with the production data (DST “perforation tests”). Baltim East (BE3) well has the most desired petrophysical characteristics in the Baltim East gas field, while, the Baltim North-1 (BN1) well showed the most favorable petrophysical parameters in the Baltim North gas field. Different fluid contacts (gas water contact GWC) were detected by integrating all reservoir pressures. The integration of different data in our present work (well logs, core measurements, and pressure data) could reduce the drilling risks and help to determine the best locations for future exploration and development, which is considered a big challenge in the petroleum industry.

## Introduction

The Egyptian Nile Delta covers nearly 60,000 km^2^ and is part of the Eastern Mediterranean basin. Two–thirds of Egypt’s gas production is provided by many hydrocarbon fields in the Egyptian Delta. The Nile Delta is a giant gas province because its discoveries represent about 82% of all Egyptian gas discoveries with reserves of 58 TCF^1^. Due to approximately 50 TCF of undiscovered potential, the Nile Delta has attracted attention. The main gas-bearing reservoirs in the Nile Delta province are developed in the sandstone interbeds of the late Miocene age (Missenian) of Abu Madi Formation, and other small discoveries are recorded in the Pliocene to Pleistocene deposits of El-Wastani Formation.

**Figure 1:**
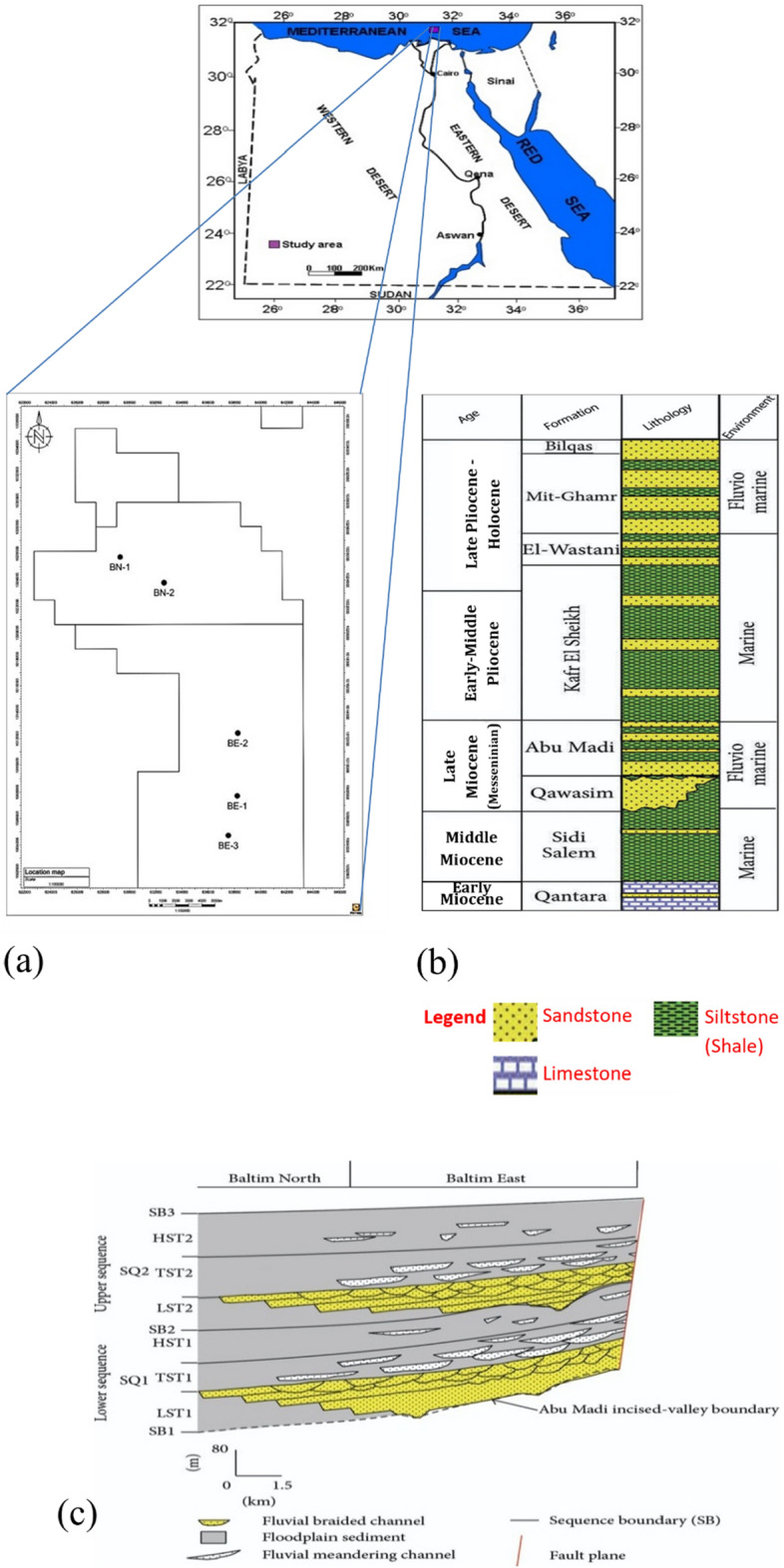
(**a**) Location map of the study area. (**b**) Lithostratigraphic column of the Nile Delta in the Baltim region of Egypt (modified after^18,22^). (**c**) The sequence stratigraphic framework cross section of Abu Madi Formation in Baltim Fields, offshore, Nile Delta, Egypt (Modified after^2,3^).

Reference^2^ shows that huge gas volumes of about (3.8 billion barrels reported in 2000) were produced from the Miocene and Pliocene Sandstone reservoirs, and more than 62 TCF are valid as proven reserves. Hence, proper characterization is significant for reducing drilling risks and improving the recovery of oil and gas^3–6^. Philips Oil Company announced in 1969 that the Abu Qir-1 well was the first gas discovery in the offshore portion^7^. In this paper, the study area covers Baltim East and North gas fields. According to the Egyptian Transverse Macerator (ETM) coordinate system, the area of investigation is located in the offshore part of the Nile Delta between latitudes 1001000 and 1037000 N and longitudes 622000 and 646000 E, as shown in Fig. 1a. About 25 km northward of the shoreline of the Nile Delta, the Baltim area is located, which is considered the northwest extension of Abu Madi, El-Qar’a main channel or Paleo-valley, covering a surface area of about 500 km^2^, with a length of 25 km and a width of 18.75 km.

The northern portion of the Abu Madi Paleo Valley area holds giant condensate gas accumulations in Baltim fields^8^. Baltim East was discovered in 1993, and production started in April 2000^9^. It includes ten wells, Baltim East—1 to 10. Good field performance as well as key workovers that occurred during the past years emphasizes the possibility of additional potential for the Baltim North area. Our present study is based on many available data sets such as well logs, pressure data, production data, and core measurements. Our main goals in this paper are to identify promising and best locations for future exploration and development, as well as to reduce drilling risks.

## Geologic settings

The Marine Nile cone is distinguished by thicker tertiary sediments composed primarily of siliciclastic rock^10^. The lithostratigraphic column of the Nile Delta in the Baltim region of Egypt is shown in Fig. 1b. The Sequence Stratigraphic Framework Cross Section of Abu Madi Formation in Baltim Fields, offshore, Nile Delta, Egypt is shown in Fig. 1c. The Basin of the Nile Delta is characterized by a large sea area regression, just like the remaining zones of the Mediterranean Sea in the late Miocene period, which allowed evaporated materials to precipitate as Rosetta Formation and fluvial to bordering sea facies as Abu Madi Formation. These were deposited in the deep incised valleys such as Abu Madi Paleo Valley^11^.

Reference^12^ showed that the incised-valley filling pattern of Abu Madi Formation is consistent with an abrupt relative sea-level fall followed by a still stand, then a progressive rise. For Abu Madi Formation reservoirs, the sections in the lower parts and upper parts consist of thick, porous sandstone, with some shale intercalations^13^. On the other hand, the top section of Abu Madi Formation consists of shale with sandstone intercalations, argues^14^. Abu Madi Formation has three zones: I, II, and zone III. Shale is the most prominent component of Zone I, but Zone II is formed of successive layers of sandstones and/or siltstones with only shale layers. Zone III, has the largest thickness, and sandstones are dominant^8^.

Reference^15^ mentioned that Abu Madi Formation is thicker in the top rock unit due to the infill sediments of the Paleo-valley “Abu Madi Paleo-valley”. As a subsurface fluvial Paleo-valley infilling, Abu Madi Formation was deposited, widening about 20 km and extending nearly 130 km from south to north. This Formation is distinguished by fluvial-deltaic sandstones and shales onlapping landward (southward) and on the flanks of the valley against the basal erosional surface (unconformity), cutting Qawasim and Sidi Salem Formations, with a thickness of about 300 m^16,17^.

The upper and lower zones’ boundaries are marked by Rosetta evaporates Formations which precipitated during the Messinian salinity crisis^18^. The Paleo Valley expands to the Baltim area in the northwest^19^. The structure of the Delta of the Nile is influenced by a lot of tectonic stages. This causes faulty arrangements, extending from the late Paleozoic to the recent^20,21^. Various trends are evident in faulty arrangements: the E–W trend (the hinge zone), the NE–SW trends (Rosetta trend), and fault movement are evident in the late Cretaceous-Early Tertiary. NW–SE trend (El Temsah trend) represents Pliocene wrench fault activity, and Baltim trend (N–S trend). The Delta of the Nile region is influenced by these above-mentioned faults, which control the reservoir trapping by joining minor faults. The first level (I) in the Baltim area is badly decayed, but the second and third levels (II and III) have shale. Our research concentrates on gas accumulations in the Baltim East and North fields in Abu Madi Sandstone Formation.

## Methodology

**Figure 2:**
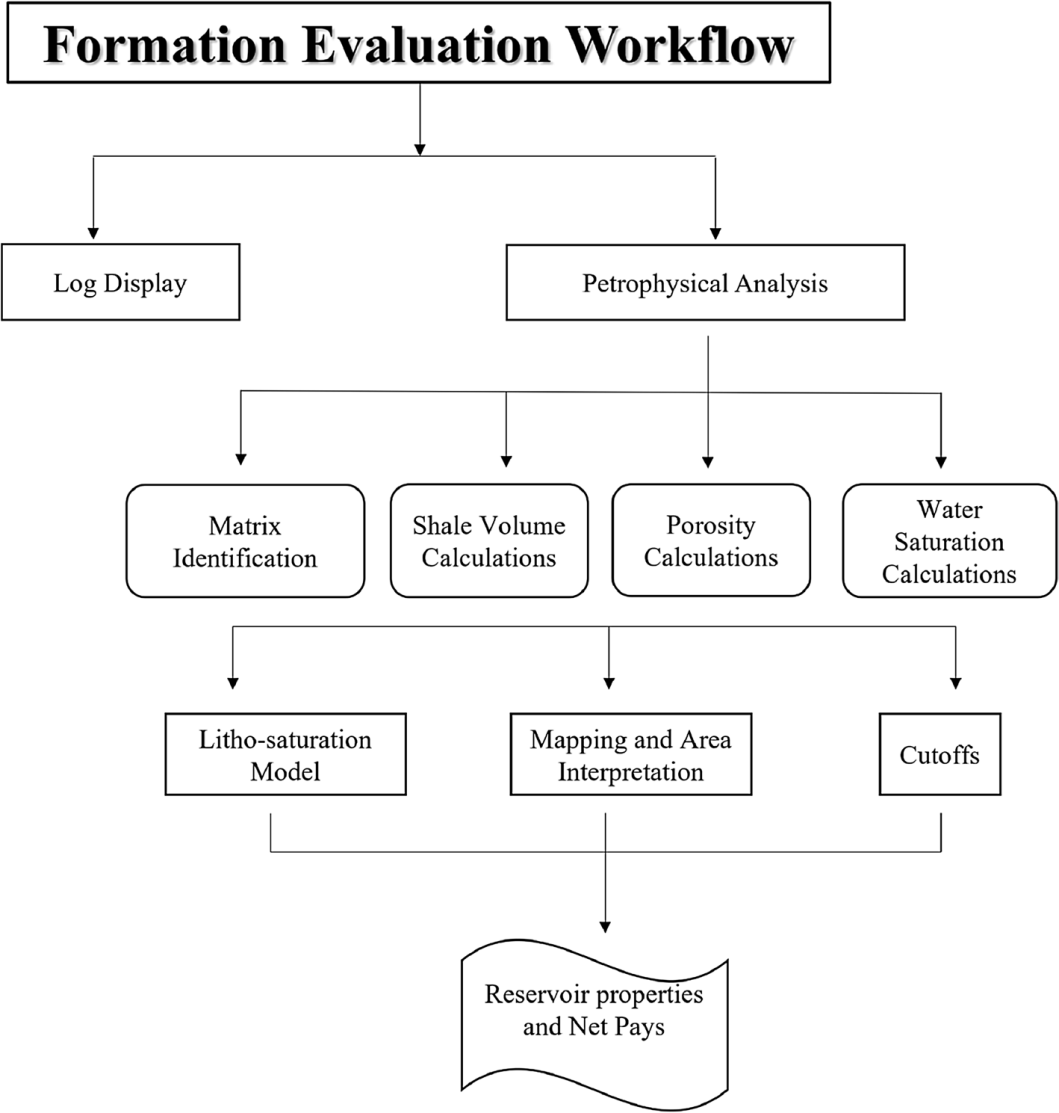
The flow chart summarizes the Formation Evaluation workflow for the studied wells.

The petrophysical characteristics of Abu-Madi reservoirs were evaluated on five wells (Baltim East 1 (BE1), Baltim East 2 (BE2), Baltim East 3 (BE3), Baltim North 1 (BN1), and Baltim North 2 (BN2) (Fig. 1a). Well logs (Gamma-ray (GR); Density (RHOB); Neutron (NPHI); PEF; Sonic (DT); Resistivity), core measurements and pressure data were used to evaluate main reservoirs and define the pay zones. First, the lithology of the main reservoir formations was identified using a combination of (Ditch Cuttings description, Core description, well log analysis, and cross plots)^22^. Then, the formation evaluation was conducted based on estimating the main petrophysical parameters: (Shale volume, effective porosity, net thickness, and fluid saturations) of the main reservoirs^23^. The core analysis of Sixty-one samples from Baltim North well’s Abu Madi Formation (level III lower) at different intervals (3765 m to 3783.20 m) was collected. Porosity, permeability, and fluid saturation derived from core laboratory measurements are also obtained. Figure 2 represents the workflow for Formation evaluation in this study.

The lateral and vertical variations of the petrophysical parameters were mapped for the study area. The average shale volume (avg. VCL) is estimated by using gamma ray (GR) well logs as expressed by Refs.^24,25^, then applying the shale correction, to accurately estimate the effective porosity (PHIE). Total porosity calculations (PHIT) are accurately estimated by using the neutron and density logs combination^26^. The Dual Model for shaly formations is used to accurately calculate the effective water saturation and then, the hydrocarbon saturation^24^.

Using Schlumberger software Techlog, the borehole environmental corrections and interpretations were carried out. Drill stem tests (perforations) at the different intervals in the BE-3 well were used to verify the presence of water and its salinity (lab analysis) which was used to compute the water resistivity (Rw) required in Archie’s water saturation equation. Pressure data was used to confirm the fluid typing.

The final results are presented in the form of litho-saturation models, cross-plots, contour maps, and pressure versus TVDSS depth plots, to identify the fluid types and contacts.

### Lithologic determination, using cross-plots

To determine accurate formation lithology, it’s very necessary to integrate the well logs responses (cross plots), with the core descriptions and ditch cuttings descriptions (mud log)^27^. Density, neutron, sonic, and gamma-ray logs are the most useful logs for this purpose. Accurate results for lithological identification can be obtained, by using Schlumberger charts and cross-plots; such as density (RHOB)–Neutron (NPHI) cross-plots and M–N cross-plots^21^. The M–N cross plot is one of the common techniques used to identify the mineral composition. It uses density, compensated neutron, and compressional sonic logs, to identify the binary and ternary mixtures of minerals. The terms M and N are defined as follows:

1 $$\mathrm{M }= \left(\Delta {\mathrm{t}}_{\mathrm{f}}-{\Delta \mathrm{t}}_{\mathrm{log}}/{\uprho }_{\mathrm{b}-}{\uprho }_{\mathrm{f}}\right)\times 0.01,$$

2 $$\mathrm{N }= {\mathrm{\varnothing }}_{\mathrm{NF}}-{\mathrm{\varnothing }}_{\mathrm{N log}}/{\uprho }_{\mathrm{b}-}{\uprho }_{\mathrm{f}},$$

where ∆t f is the formation fluid transit time (µs/ft), ∆t log is the transit time read from the sonic log (µs/ft), ${\uprho }_{\mathrm{b}}$ is the bulk density read from density log (g/cc), ${\uprho }_{\mathrm{f}}$ is the formation fluid density (g/cc), ${\mathrm{\varnothing }}_{\mathrm{NF}}$ is the fluid neutron porosity (decimal), ${\mathrm{\varnothing }}_{\mathrm{N}}$ is the neutron porosity read from neutron log (decimal).

**Figure 3:**
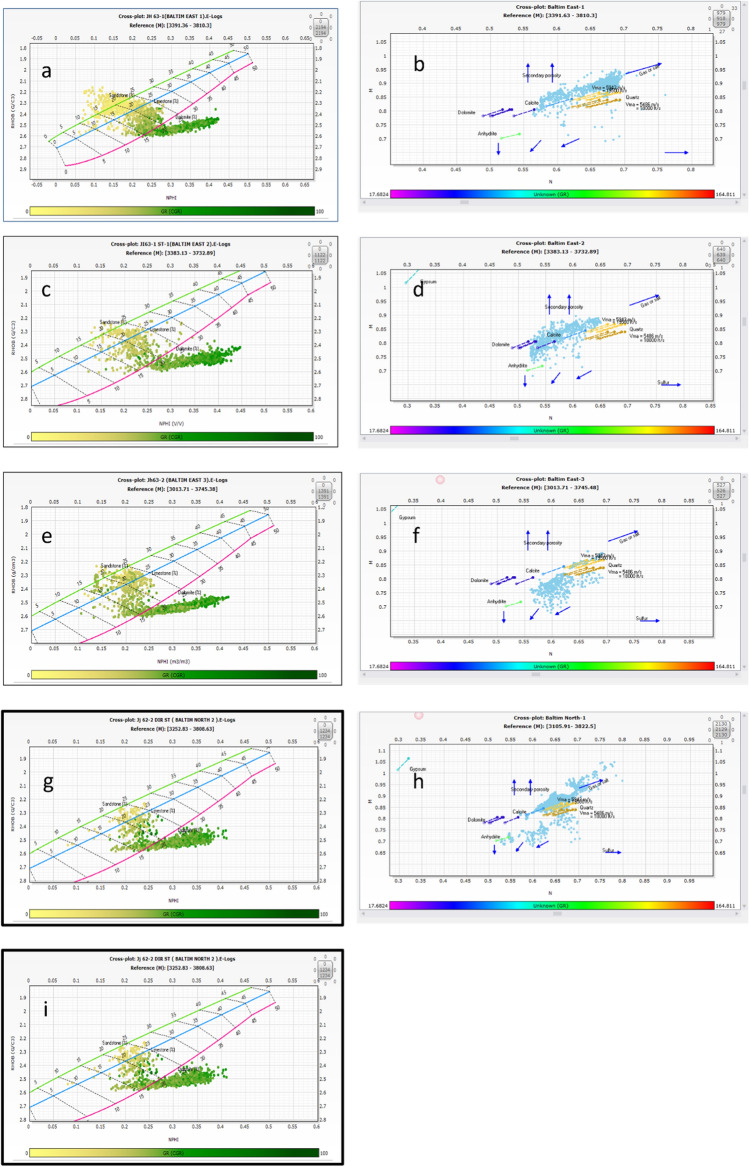
(**a,c,e,g,i**) Are the (RHOB-NPHI) cross plots of Abu Madi Formation of BE1, BE2, BE3, BN1, and BN2 wells, and (**b,d,f,h**) are (M–N) cross-plots of Abu Madi Formation of BE1, BE2, BE3, and BN1 wells.

In the present study, various charts and cross plots were applied, to obtain the types and minerals of each lithologic component of Abu Madi Formation. Generally, Abu Madi Formation lithology was focused, which was nearly homogenous in all five studied wells. To make a correct interpretation of Abu Madi lithologies, we used the different log combinations to create: (a) Density (RHOB log) − Neutron (NPHI log) cross plot. (b) Density (RHOB log), Neutron (NPHI log), and sonic (DT log) with density and neutron logs, to obtain an M–N cross-plot (tri-porosity cross plot), as shown in Fig. 3.

### Litho-saturation model for studied wells

It serves as a reliable petrophysical interpretation model. It explains the division of the formation as depicted in Fig. 11 and is presented as a log in Fig. 12. Shale volume, effective porosity, and bulk water volume are the three computed petrophysical characteristics that make up the bulk rock volume. Along with shale volume and the degree of hydrocarbon content saturation, our model will make the percentage of matrix volume clear. The volume of the matrix can be broken down into its component parts, such as, sandstone, limestone, dolomite, etc.^4–6,28,29^.

### Area interpretation maps and correlation

The average values of the Abu Madi reservoir’s petrophysical parameters were constructed based on Table 1 to show the lateral variation within the studied sandstone reservoirs.

**Table 1:** The reservoirs and pays summaries for the Abu Madi and Qawasim Formations in all studied wells.

Well	Formation	Top (m)	Bottom (m)	Gross thickness (m)	Net reservoir (m)	N/G	Net. pay (m)	Avg. PHIE (%)	Avg. Sw (%)	Avg. Sg (%)	Avg. Vcl (%)
Baltim East 1	Abu Madi	3423.5	3758	334.5	131.5	0.393	46	19	42	58	5
	Qawasim	3758	3803.5	45.5	6	0.132	Zero	18	93	7	2
Baltim East 2	Abu Madi	3465	3640	175	48.5	0.277	28	17	41	59	6
	Qawasim	3640	3711	71	2	0.028	Zero	19	85	15	4.6
Baltim East 3	Abu Madi	3513	3725	212	62	0.292	40	21	42	58	1
Baltim North 1	Abu Madi	3564	3803	239	83	0.347	61	18	40	60	12
Baltim North 2	Abu Madi	3607	3795	188	25.5	0.136	6	15	60	40	22

### Core data analysis

Core data are a very important calibration tool for litho-saturation plots. In the present study, the core measurements helped in the effective porosity and water saturation calibration. There are three wells containing core data at level III main (BE1, BE3, and BN2), while the only well with core data at level III lower is BN1. From Fig. 12 there is a good match between the effective porosity from CPI and the core effective porosities in levels III main and lower. 61 cores from the BN1 well were utilized as a reference to the wireline logs^30–32^.

### Formation fluid type and fluid contacts

The static pressures in virgin reservoirs are not affected by the withdrawal of fluids, so the observed gradients show and represent the original fluid density. The original fluid contacts can be reflected and represented by the “breaks”, where the slope changes in the gradient^33,34^. Different types and contacts of Formation fluid have been explained by different authors, e.g.,^14,33,35–38^.

## Results

### Lithologic determination, using cross-plots

The cross-plots in Fig. 3a,c,g indicate that sandstone, calcareous shale, and siltstone are the primary lithologies of Abu Madi Formation in the BE1, BE2, and BN1 wells, with a notable presence of calcareous materials. Most data points on these plots, colored yellow and orange, cluster in the upper left side, indicating the presence of gas.

In contrast, Fig. 3e,i show that calcareous shale, sandstone, and siltstone dominate in the BE-3 and BN-2 wells, with data points from Abu Madi reservoirs aligning along the sandstone line. Some points are near the limestone and dolomite lines, confirming the presence of calcareous materials, and gas presence is indicated by shifted points in the upper left side.

The M–N cross-plots in Fig. 3b,d,f,h corroborate the density–neutron cross-plots and reveal lithologic components. These plots show sandstone lithology as points in the quartz part, calcareous minerals around the calcite, and the presence of calcareous materials intercalated with shale near anhydrite and dolomite areas. Gas saturation is indicated by arrows and shifted points on the upper right side, while sandstone content is represented by points along the sandstone line in the quartz matrix zone.

In summary, the predominant lithology vertically through the Abu Madi Formation is sandstone transitioning to calcareous lithology with shale and siltstone. These findings align well with core and mud log lithologic descriptions, providing consistency in the analysis.

### Litho-saturation model for studied wells

#### Litho-saturation model for BE-1 well

**Figure 4:**
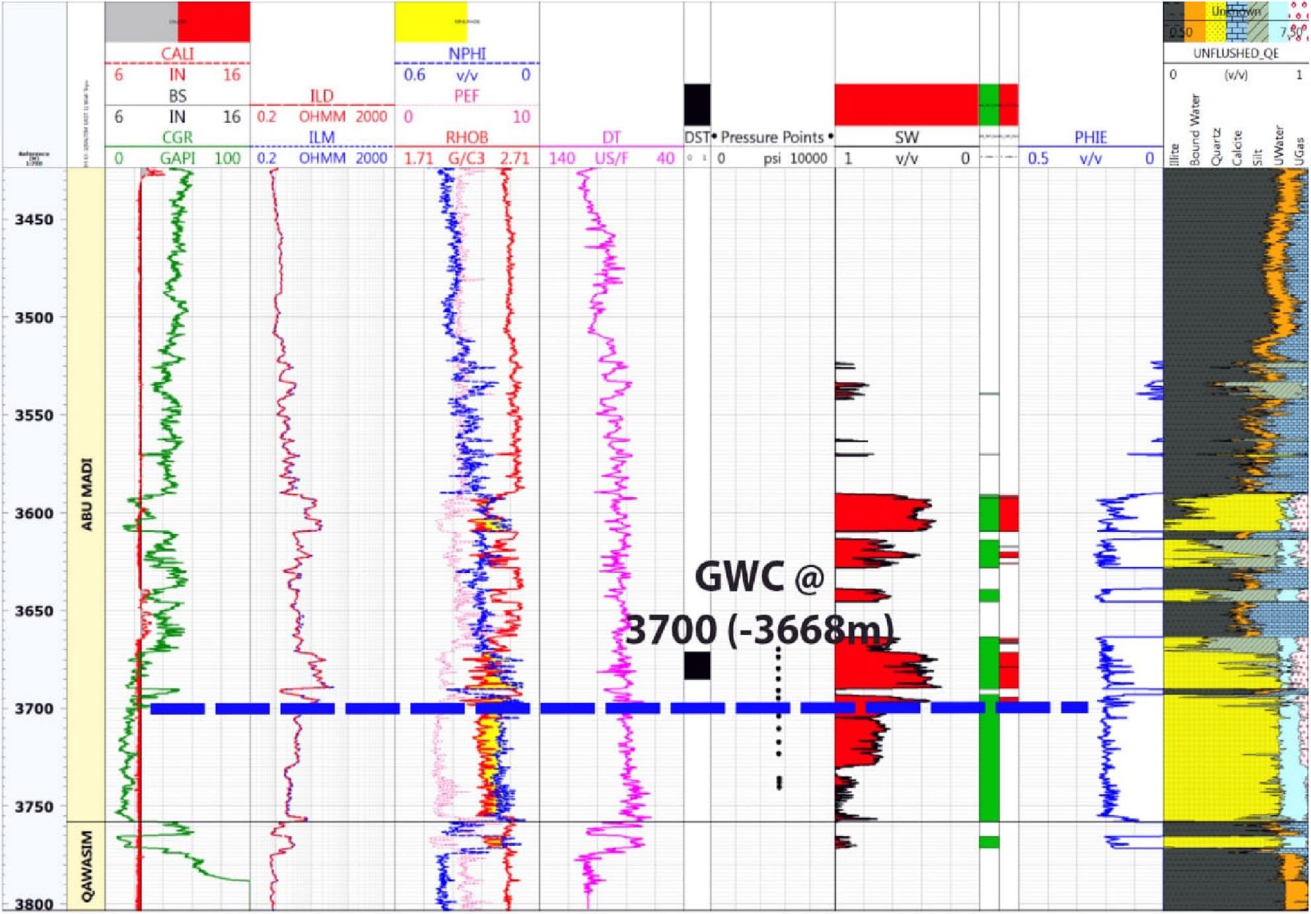
Computer processed interpretation for the Baltim East-1 well.

Utilizing cutoff values of Vsh (shale volume) at 40%, Ø (porosity) at 8%, and Sw (water saturation) at 60%, we find that in the Abu Madi Formation of the BE-1 well, there is approximately 46 m of net pay. This interval exhibits an average shale volume of 5%, an average effective porosity of 19%, and an average water saturation of 42%. In contrast, the net reservoir thickness within the Qawasim Formation is only around 6 m, and it does not contain any pay zones. Figure 4 depicts the litho-saturation model for the Baltim East-1 well.

#### Litho-saturation model for Baltim East-2 well

**Figure 5:**
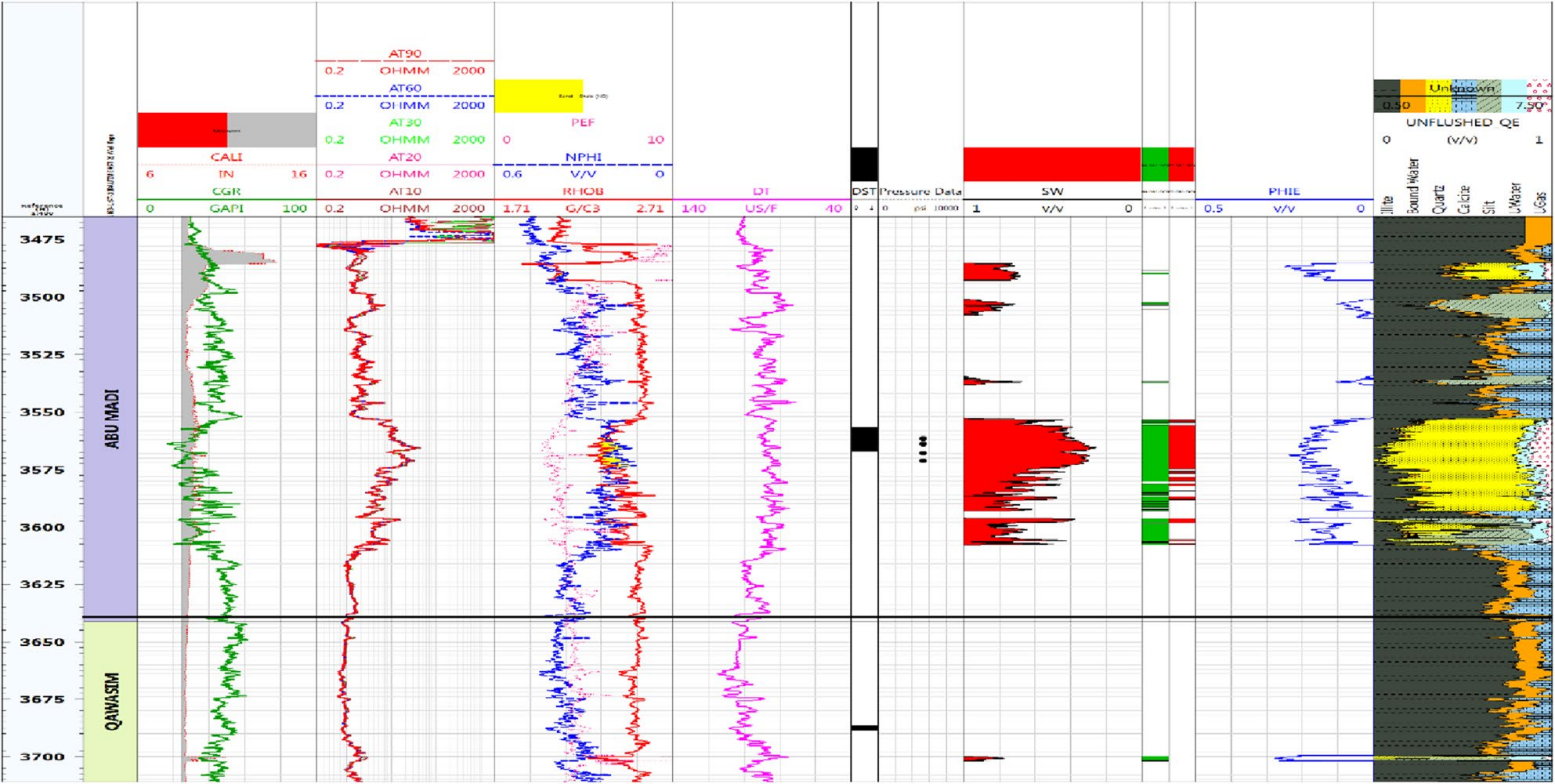
Computer processed interpretation for BE-2 well.

As shown in Fig. 5, the Abu Madi Formation prominently consists of thick sandstone layers, while the Qawasim Formation yields a net reservoir thickness of approximately 2 m, devoid of any productive zones. It is worth noting that during a Drill Stem Test (DST) conducted within the depth interval of 3556 m to 3567 m, the results indicated the presence of both gas and condensate.

Upon implementing specific criteria, including a shale volume cutoff of 40%, a porosity threshold of 8%, and a water saturation limit of 60%, the net pays within the Abu Madi Formation measures around 28 m. Within this zone, the average shale volume is 6%, the average effective porosity is 17%, and the average water saturation is 41%.

#### Litho-saturation model for Baltim East-3 well

**Figure 6:**
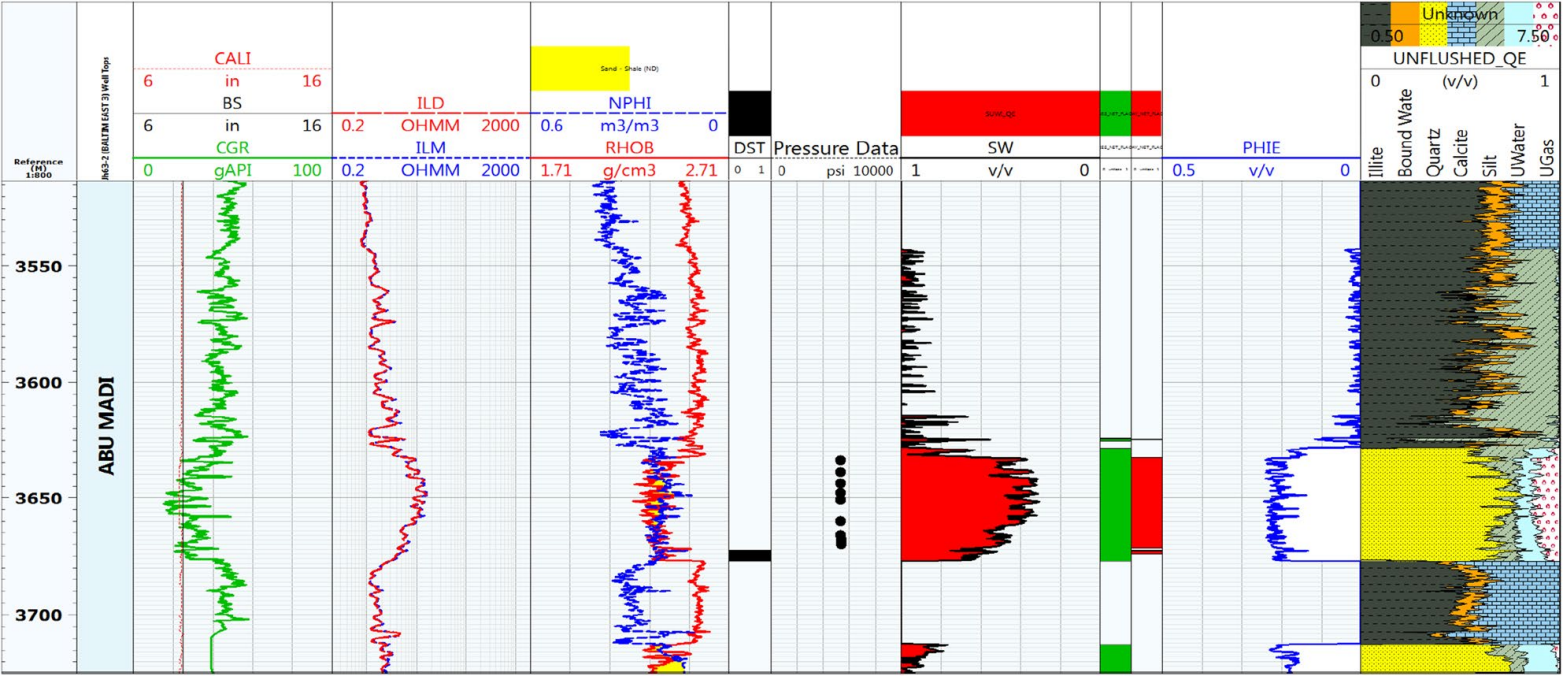
Computer processed interpretation for BE-3 well.

As illustrated in Fig. 6, the log interpretation reveals the prevalence of thick sandstone layers within the depth range of approximately 3630 m to 3675 m. It’s worth noting that a Drill Stem Test (DST) was conducted in the interval from 3672.5 to 3677 m to investigate the presence of water and its salinity through laboratory analysis. The outcome of the DST indicated the presence of water with a salinity level of 14 KPPM.

Utilizing specific parameters provided by the production and core analysis (including a salinity of 14 KPPM, m = 1.69, n = 2, and a = 1), and applying predetermined cutoff values (shale volume ≤ 40%, porosity ≥ 8%, and water saturation ≤ 60%), we can determine that the net pay zone within the Abu Madi Formation spans approximately 40 m. Within this zone, the average shale volume is minimal, standing at just 1%, while the average effective porosity is a notable 21%, and the average water saturation is approximately 42%. It's important to note that drilling was halted at a depth just before reaching the Qawasim Formation.

#### Litho-saturation model for Baltim North-1 well

**Figure 7:**
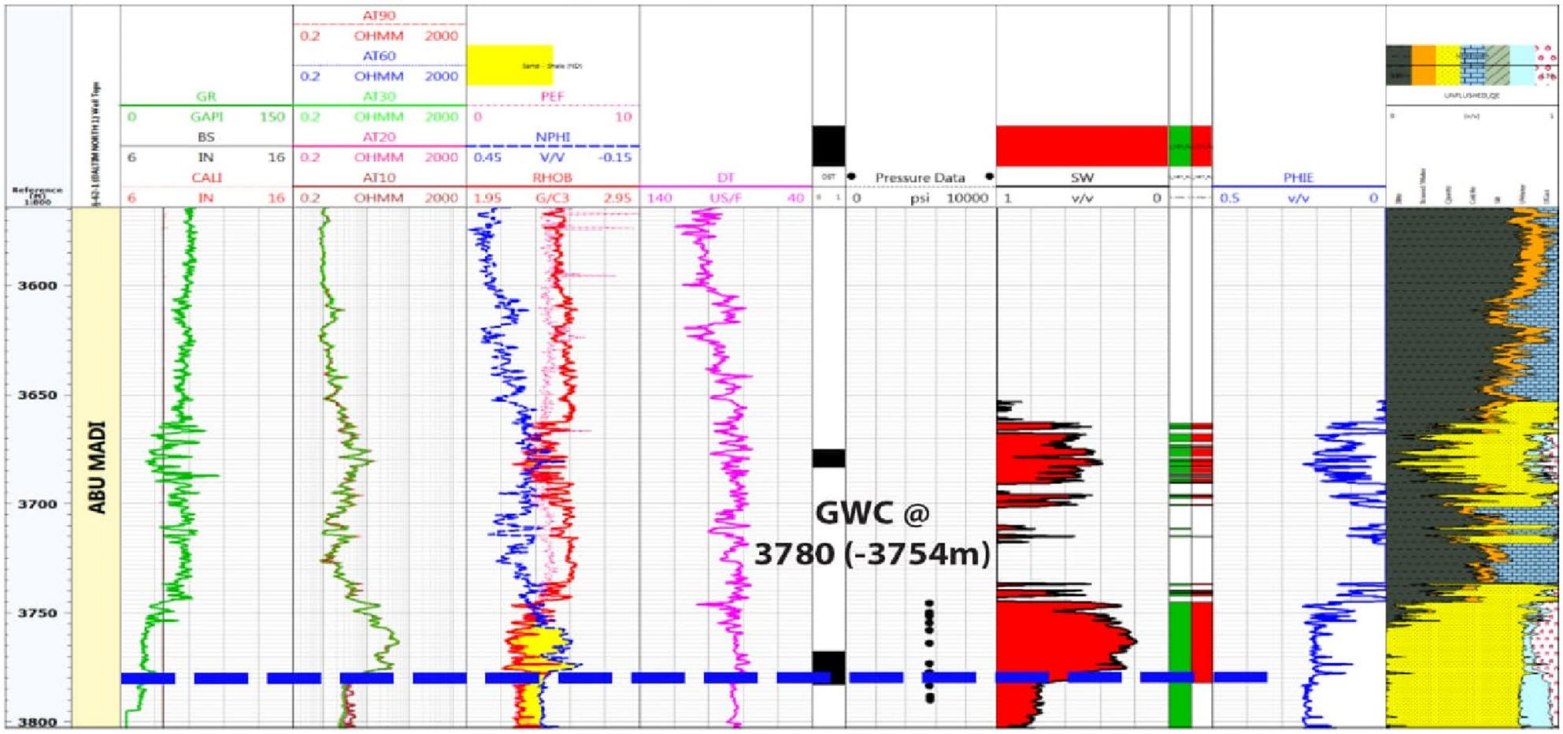
Computer processed interpretation for BN-1 well.

Figure 7 displays the Continuous Pressure Index (CPI) for the Baltim North-1 (BN1) well, highlighting the substantial presence of thick sandstone within the Abu Madi Formation zones, particularly in levels III Main and lower III. The figure also presents results from two Drill Stem Tests (DSTs) conducted at different depths to confirm the existence of gas in the Abu Madi sandstone zones. The upper DST conducted between 3674 and 3683 m, and the lower DST, between 3766.7 and 3782m, both confirmed the presence of gas and condensate and identified the gas–water contact (GWC) at 3780 m (3754 m TVDSS). Analyzing production and core data using established criteria, the study determined a net pay thickness of approximately 61 m, characterized by an average shale volume of 12%, an average effective porosity of 18%, and an average water saturation of 40%. Furthermore, the depth-pressure plot for the BN1 well validated the GWC's presence at a depth of 3754m (TVDSS), adding crucial information to the reservoir evaluation.

#### Litho-saturation model for Baltim North-2 well

**Figure 8:**
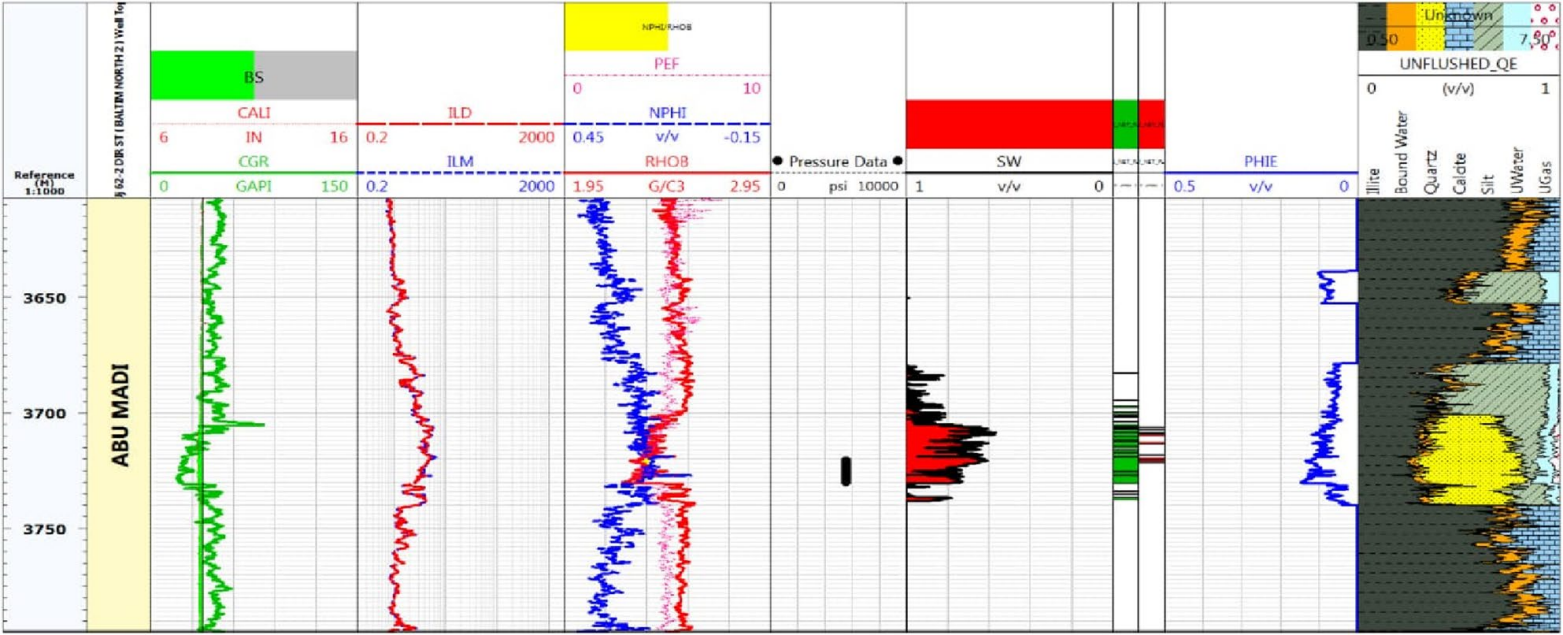
Computer processed interpretation for BN-2 well.

The BN-2 well’s CPI (Fig. 8) demonstrates that the well is characterized by the predominance of thick sandstone, with a net reservoir of about 25.5m. Well, TD came before entering Qawasim Formation. There is no DST taken in the BN-2 well. By using the production and core data parameters and applying the cutoffs (shale volume 40%, porosity 8%, and water saturation 60%), the net pay (Abu Madi Formation) is about 6 m, with an average shale volume of 22%, average effective porosity of 15% and average water saturation 60%.

### Area interpretation maps

The summaries of the Abu Madi reservoirs and pays were discussed and analyzed as follows.

**Figure 9:**
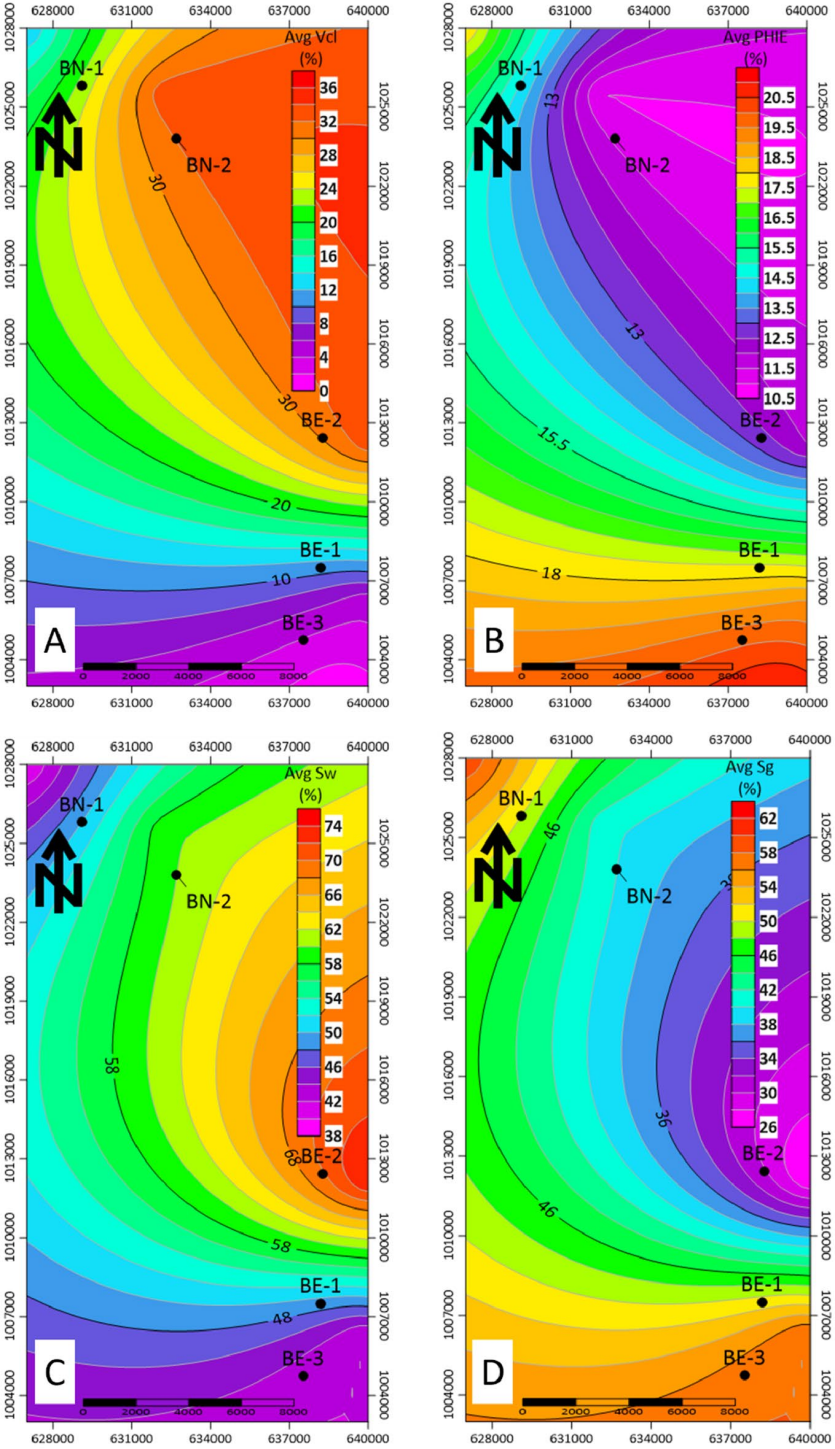
Contour maps of the average (**a**) Shale volume, (**b**) effective porosity, (**c**) water saturation and (**d**) gas saturation.

Shale volume is a significant parameter and a very important indicator of the reservoir quality, so shale volume maps are constructed in this study, to illustrate and reflect the reservoir quality and its characteristics. Shale volume generally increases towards the north at BN1 and BN2 wells, as shown in Fig. 9a, with values of around 12% and 22%, respectively. The values of (VCL) in the Baltim East wells are lower than the shale values in the Baltim North wells. The average percentages of the shale volume at BE-1, BE-2, and BE-3 wells are around 5%, 6%, and 1%, respectively. Shale volume maps showed that Baltim East wells have lower values of shale content than Baltim North wells.

Effective porosity calculations are obtained by applying the shale correction to the total porosity calculations. The average effective porosity map shows BN-1 and BN-2 wells, with values of around 18% and 15%, respectively. At Baltim East wells, BE-1, BE-2, and BE-3 wells, have nearly values of 19%, 17%, and 21%, respectively. These average effective porosity values are calculated for a well’s pay and reveal that BE-3, BE-1, and BN-1 have higher values in the studied wells, as shown in Fig. 9b.

The main target of this study is to calculate and evaluate the hydrocarbon saturation, so the lateral distribution map of gas saturation for the five studied wells is presented. Figure 9c shows the water saturation distribution for the Abu Madi reservoir in the wells under investigation and Fig. 9d shows the gas saturation map for the Abu Madi reservoir in the wells under investigation. This figure reveals that the average gas saturation (Sg = 1 − Sw) is 58%, 59%, and 58% for BE-1, BE-2, and BE-3, respectively. At the Baltim North field, the average gas saturation values that are calculated for BN-1 and BN-2 pay are nearly 60% and 40%, respectively. It’s shown that the BN-2 well has the minimum gas saturation value; this decrease may be due to the high value of shale and low effective porosity value.

**Figure 10:**
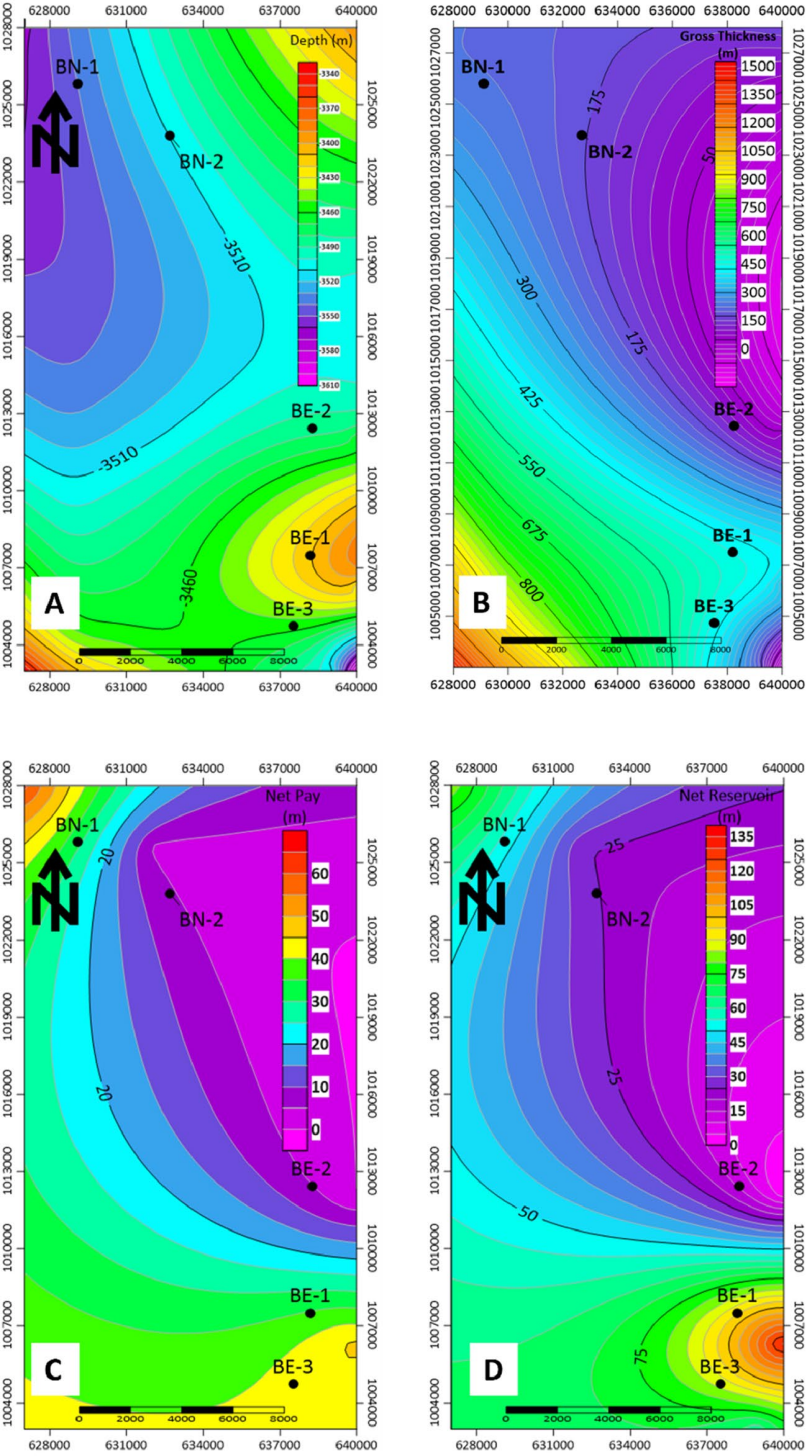
(**a**) Contour map of depth to Abu Madi top, (**b**) contour map of the Gross thickness, (**c**) contour map of the Net reservoir of Abu Madi Formation, and (**d**) net pay (effective thickness) of Abu Madi Formation.

Figure 10a the depth to Abu Madi Top for Abu Madi Formation, Fig. 10b shows the gross thickness, Fig. 10c shows Abu Madi Formation net reservoir, and Fig. 10d shows the net pay (effective thickness) of Abu Madi Formation.

### Correlation

**Figure 11:**
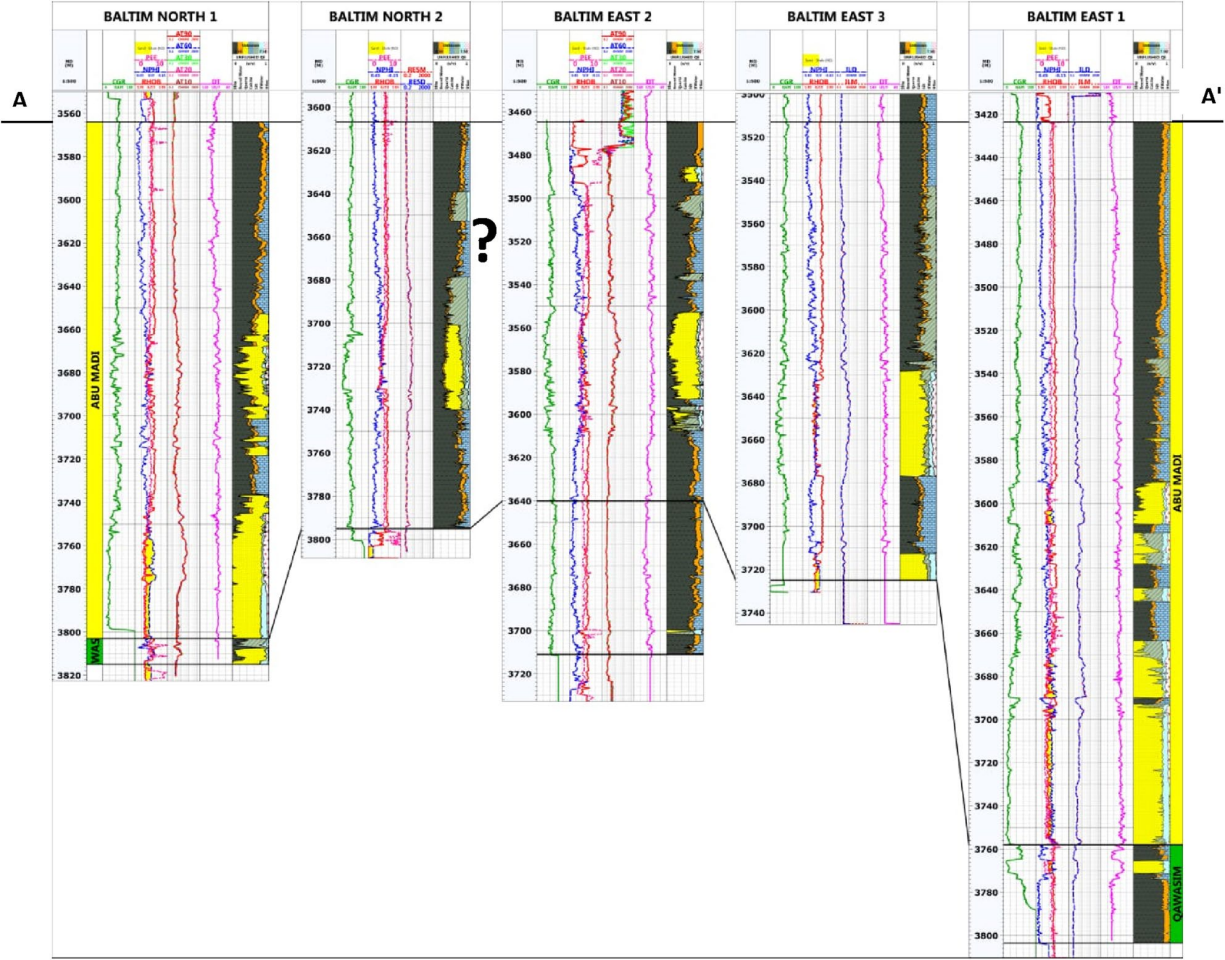
Lithostratigraphic petrophysical correlation profile (AAʹ) of BN1, BN2, BE2, BE3, and BE1 wells, as flattened on Abu Madi Formation top using the information inferred from available logs. (?) indicates uncertainty due to the existing significant gap).

The correlation panel in Fig. 11 shows the lateral variation of petrophysical characteristics. Reference^39^ showed that the study of these parameters could be important in the lateral variation indication and the factors controlling them, which may be either stratigraphic or structural, or both.

The well correlation demonstrates the following:

1. Net reservoir has decreased in the BN2 (25.5 m) and BE2 (48.5 m) wells. This decrease may be due to unconformity surface or fault presence, so something is missing in Abu Madi (level III lower) Formation of both wells^20^ because Abu Madi Formation is only penetrated by these two wells, the thickest Abu Madi net reservoir is represented by BE1 = 131.5 m and BN1 = 83 m.
2. The significant difference in depth to Abu Madi Formation tops between Baltim East wells (depth to the Abu Madi Top at BE1 = 3423.5 m) and Baltim North wells (depth to Abu Madi Top at BN2 = 3607 m), the great difference in depth may be caused by E–W trending faults at the northern and southern margins of the study area. Due to the main faults that dip towards the north and many structures (fractures and minor faults) in between, the Baltim area, it is shown that the depth of the Abu Madi Formation top increases towards the north direction and decreases towards the south direction as a result of the dipping of Formation towards the north^8,20^.

### Core data analysis

**Figure 12:**
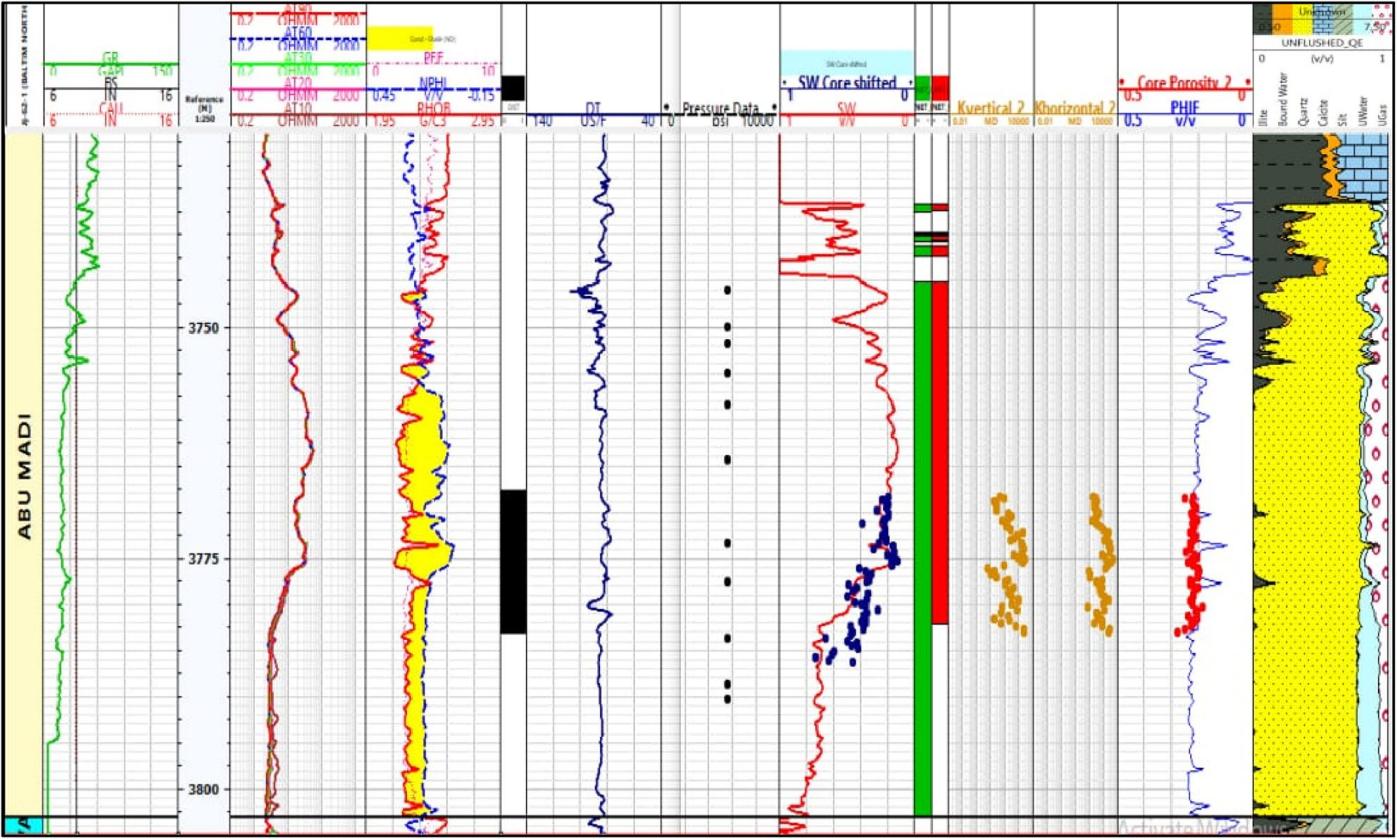
Illustrates BN1 well log data and core data plot.

Core samples were extracted from the Baltim N-1 well (Fig. 12), and routine core analysis revealed key reservoir characteristics, focusing on two main types of reservoir qualities. Type I characterized as Macro pores displayed an average nitrogen permeability of 623mD and a helium porosity of 21.7%. Type II, classified as Mega pores, exhibited an average nitrogen permeability of 2479mD and a helium porosity of 23.5%. The permeability of rocks is influenced by various factors such as pore geometry, particle size, impurities in water, void rock ratio, saturation level, clay content, and diagenesis. The petrophysical properties data from the core, specifically the porosity and permeability measurements (K-phi data), indicate that this reservoir possesses high-quality reserves and favorable fluid flow properties. Notably, depth intervals from 3765.00 to 3768.60 to 3772.50 to 3778.80 m are characterized by good reservoir quality with Macro pores, while intervals from 3768.90 to 3772.20 m and 3779.10 to 3783.00 m exhibit very good reservoir quality with Mega pores.

### Formation fluid type and fluid contacts

**Figure 13:**
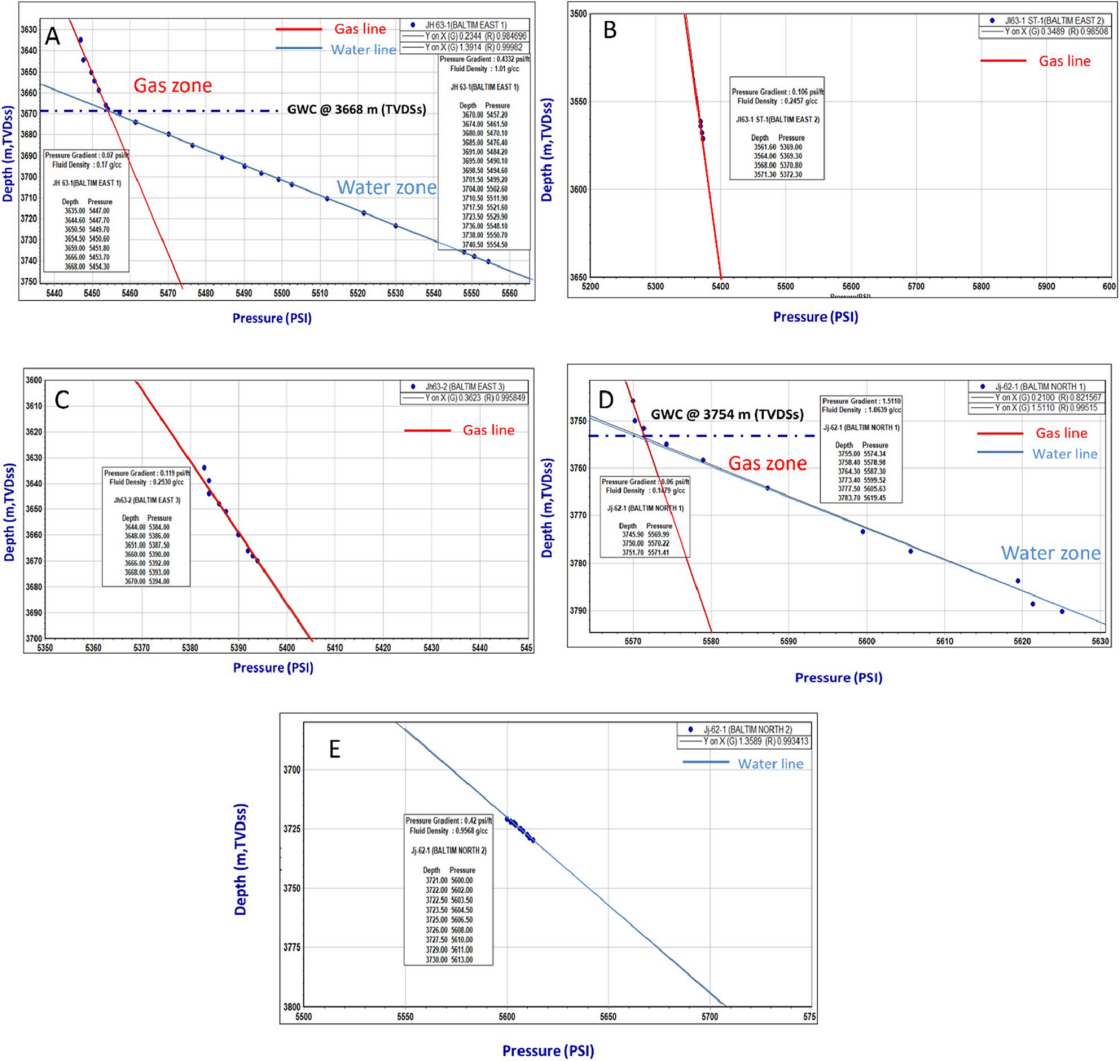
Pressure plots for Abu Madi Formation in BE-1, BE-2, BE-3, BN-1, and BN-2 wells, illustrate the gas and water gradients and GWC.

The data on formation pressure in the Baltim East and North wells provide crucial insights into fluid types, fluid contacts, and reservoir conditions. These findings are essential for reservoir characterization and production management. In the Baltim East-1 well (Fig. 13a), a gas–water contact (GWC) was identified at a depth of 3668 m TVDSS. The pressure gradient suggests a fluid type of gas condensate, with a low-salinity formation water gradient. This GWC aligns with open-hole log data^37^. In the Baltim East-2 Well (Fig. 13b), no GWC was observed due to all reservoirs being above the main contact. The pressure gradient indicates a gas and condensate fluid type, as corroborated by production data. GWC could be approximately located using open-hole logs.

Moving to the Baltim East-3 well (Fig. 13c), again, no GWC was observed within the gas pay, and the pressure gradient supports a gas and condensate fluid type. Laboratory analysis of the perforation interval confirmed the presence of water. In the Baltim North-1 well (Fig. 13d), a GWC was observed at 3754 m TVDSS, matching the open-hole log data. The pressure gradient indicates a gas and condensate fluid type, confirmed by DST data.

Finally, in the BN-2 well (Fig. 13e), within the depth interval of 3721 m to 3730 m TVDSS, the pressure gradient suggests the presence of water, particularly in the water leg below the GWC and the transition zone between the gas column and water leg.

Combining these pressure plots with Litho-Saturation models, Drill Stem Test (DST) or Perforation Test results, and other data, one can accurately define fluid types and fluid contacts in the Baltim East and North wells. Additionally, the pressure data can aid in understanding reservoir depletion and its impact on water saturation over time. These comprehensive pressure analyses are crucial for effective reservoir management and optimizing hydrocarbon production in the studied wells.

## Discussion

As shown in Figs. 4 and 7, Abu Madi Formation whole section is penetrated in the BE1&BN1 wells, Abu Madi Formation’s gross thickness is 334.5 m in the BE1 well and 239 m in the BN1well (Fig. 10b), representing the largest thickness in the five studied wells. In these two wells, the gas and water zones could be detected, then the GWC is also delineated. There is a good cross-over between the neutron and density logs, that occurs in the gas zone, with high resistivity, as well a small neutron–density cross-over occurs in the water-saturated zone with nearly low resistivity values. A Direct approach for the most important petrophysical parameters’ evaluation (BVH, BVW, Vsh, and Øeff) at each depth in each well, can be given by using the litho-saturation models^40–42^. The litho-saturation plots are constructed to give a complete view of the lithological analysis (Vsh and matrix), Øeff, and the internal resolution of the available saturation, including gas and water saturations.

The correlation of wells proved that the thicker sandstone layers in the Abu Madi reservoir combined with the better petrophysical characteristics of higher effective porosity, lower shale value, and higher gas saturation produced a good petrophysical set-up for the investigated Abu Madi section in the central portion of the Paleo-valley area.

It is shown from the pressure plots of our five studied wells (Fig. 13) that the available pressure data in our present study illustrated that only two wells (BE-1 and BN-1) have data penetrated both the gas column and water leg. The BE-2 and BE-3 wells’ pressure data penetrated only the gas column and didn’t penetrate the water leg. The pressure data for BN-2 didn’t penetrate the gas column; it penetrated only the transition zone (gas–water transition zone) and the water leg.

**Figure 14:**
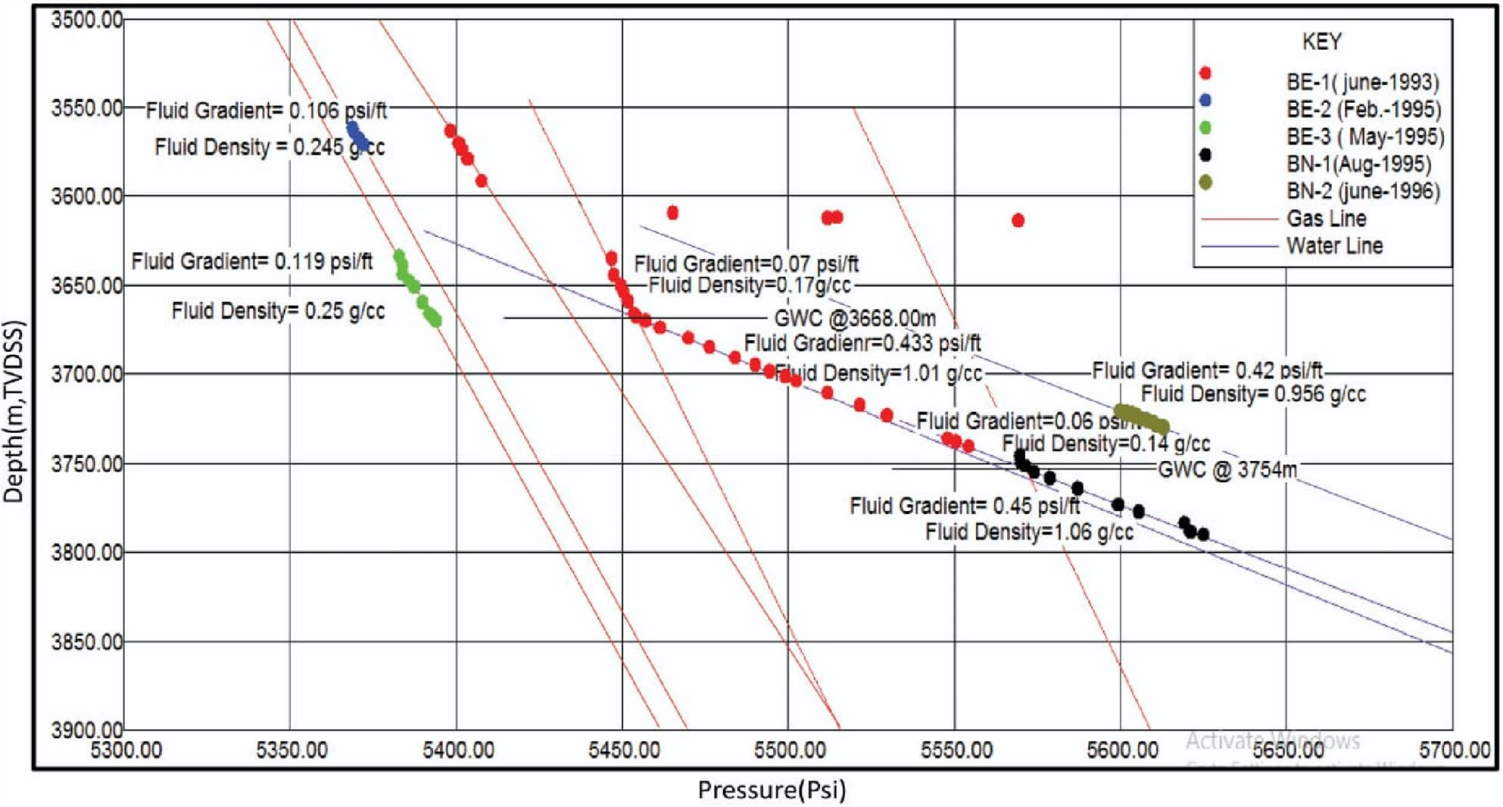
Pressure (psi) versus depth (m, TVDss) plots for the five studied wells.

Figure 14 shows the Baltim East 1 Well which is the first exploratory drilled well in our studied wells. There is a low decrease in the pressure values between BE1, BE2, and BE3 (Baltim East gas field, for example, at depth (TVDSS) = 3560 m pressure values are 5400 psi and 5375 psi for BE1 and BE2 respectively. For BE3, which was the third drilled well in the Baltim fast field (May 1995), it’s noticed that the pressure data values are about 5380 psi for a depth = 3650 (m, TVDSS), which is also considered a low decrease or low depletion.

In the Baltim North field, the pressure values also decreased within about 1 year for the BN1 well. The pressure was 3575 psi at a depth of 3750 m. The pressure data for BN2 is also considered a good indication of the low depletion that occurred in the Baltim North gas field. Two gas water contacts are represented on the (depth vs. pressure) plot, at BE1 and BN1 wells at different depths, which may be caused by the dipping of the layers towards the north. The great difference in GWC between BE1 and BN1 may give a significant indication of the presence of gas-condensate within this depth interval (3668m: 3754m). Finally, a low decrease in pressure values (low depletion) indicates less connection between the five wells that could be caused structurally or stratigraphically.

## Conclusion

In conclusion, the comprehensive petrophysical analysis and evaluation of the Abu Madi Formation in the Baltim gas field reveal significant hydrocarbon potential across the studied wells. The petrophysical characteristics studied through wire-line logs and core data provide valuable insights into the reservoir’s quality and distribution.

1. *Reservoir characteristics* The net pay thickness in the Abu Madi Formation varies across the wells, with BE-1 having approximately 46 m, BE-2 at 28 m, BE-3 at 40 m, BN-1 at 61 m, and BN-2 at 6 m. These substantial pay intervals suggest the presence of considerable hydrocarbon accumulations within the formation.
2. *Effective porosity* The average effective porosity falls within the range of 15% to 21%, which is indicative of good reservoir quality. Higher effective porosity values are particularly notable in BE-3, suggesting favorable petrophysical performance in that well.
3. *Shale volume* The shale volume (Vsh) ranges from 1 to 22%. Lower shale volume, as observed in the central portion of the paleo Valley, contributes to better petrophysical quality and performance, making it a promising area for future exploration and development.
4. *Hydrocarbon saturation* Hydrocarbon saturation varies from 40 to 60% in the studied wells, further indicating the presence of commercial hydrocarbon accumulations. Gas saturation of around 60% in BN-2 is noteworthy, though it is accompanied by higher water saturation.
5. *Pressure data* Pressure data analysis reveals that the wells contain gas condensate, as indicated by pressure gradients ranging from 0.06 to 0.12 psi/ft. These pressure gradients align well with the litho-saturation models and are supported by production data from DSTs, reinforcing the assessment of the gas-condensate nature of the reservoir.
6. *Future prospects* The central portion of the paleo Valley emerges as a highly promising area for future exploration and development due to the presence of thicker Abu Madi sandstone intervals with higher average effective porosity and lower shale volume. Additionally, the southern part of the gas field, characterized by a low depletion rate as evident from pressure plots, remains favorable for ongoing production.
7. *Consistency with core and pressure data* It is important to highlight that the petrophysical analysis results align consistently with core data and pressure data, providing a robust basis for the assessment of the Abu Madi Formation’s hydrocarbon potential.

In summary, the combination of petrophysical analysis, core data, and pressure data strongly supports the conclusion that the Abu Madi Formation within the Baltim gas field possesses substantial hydrocarbon potential. The findings indicate favorable reservoir characteristics, making it a promising area for further exploration, development, and continued hydrocarbon production.

## Data Availability

Data can be requested from Mr. Ahmed Ismail Mahmoud the coauthor of this research.
